# Social media network analysis of Smallholder livestock farming communities in the United Kingdom

**DOI:** 10.1016/j.heliyon.2023.e23265

**Published:** 2023-12-03

**Authors:** Samuel Munaf, Kevin Swingler, Franz Brulisauer, Anthony O'Hare, George Gunn, Aaron Reeves

**Affiliations:** a Division of Computing Science and Mathematics, University of Stirling, Stirling, United Kingdom; b Centre for Epidemiology and Planetary Health, Department of Veterinary and Animal Sciences, Northern Faculty, Scotland's Rural College (SRUC), Inverness, United Kingdom; c SRUC Veterinary Services, Scotland's Rural College (SRUC), Inverness, United Kingdom; d Centre for Applied public health research, RTI international, Raleigh, NC, USA

**Keywords:** *Smallholdings*, *network analysis*, *social media*, *Livestock farming*, *Information dispersion*

## Abstract

The creation of targeted policies and actions to help small-scale livestock keepers and reduce the risks associated with disease outbreaks in this sector is hampered by the scarcity of information about smallholder farmers. Smallholders play a crucial part in disease outbreaks containment, hence there is a need for better monitoring methods that take this population into account while gathering data. According to the literature, these communities frequently use social media as a channel for communication and information exchange. In this study we conducted social network analysis of an influential smallholder within the UK and visualised the user follower network. Additionally, we performed influential user analysis, Twitter user categorisation, and community detection to uncover more insights into the livestock farming networks. Our findings reveal distinct communities within the smallholder farming sector and identify influential users with the potential to impact information dissemination and animal health practices. The study also highlights the role of community structure in surveillance and control of animal diseases and emphasises the need for further research to refine our understanding of these communities and their unique characteristics. This work contributes to the growing body of literature on small-scale livestock farming in the UK and underscores the importance of incorporating smallholder communities into disease surveillance and control efforts.

## Highlights

• Used Twitter data to conduct a network analysis of smallholder farmers.
• Detected key users and community structures in the network.
• Smallholders' spatial distribution was revealed through geolocation mapping.
• Communication and disease management strategies can be tailored based on findings.
• Potential of social media data demonstrated in agricultural research.

## 1 1/. Introduction

Smallholder farming is critical to global food security, local economies, and rural lifestyles [1]. The importance of small-scale farming in the agriculture sector is not limited to developing nations, but also present in countries like the United Kingdom. In the United Kingdom, they contribute considerably to the pig and poultry industries, offering diversified, sustainable, and locally sourced goods [2]. Small farms (less than 50 acres) that provide a primary or secondary income for families are significantly different from their counterparts in developing nations in terms of socio-economic aspects due to diverse local economies, infrastructure, and governmental support [3]. Understanding their social networks, interactions, and concerns is critical for improving animal health, disease surveillance, and management tactics [4,5].

Animal and Plant Health Agency (APHA) doesn't require smallholders to register their flock if they own less than fifty poultry animals, regardless of the species [6]. Registering small flocks of poultry with a governmental agency is not obligatory but can be done voluntarily. Additionally, the limited interaction with local authorities exacerbates this problem and further complicates the challenges faced by public health surveillance authorities [7]. Several initiatives are in place to aid these communities in the 10.13039/100007472United Kingdom (UK) through government support, including grants for land improvement, assistance for agritourism, and training programmes [8]. Nevertheless, there are obstacles when it comes to accessing markets, as smaller farms face competition from larger ones in terms of scale and efficiency.

Despite these limitations, a rich tapestry of diversity exists within the United Kingdom (UK) agricultural sector, with variations in the types of smallholders, who all contribute to bolstering food security, sustaining rural economics, preserving biodiversity and cultural traditions. Through their established methods, traditional smallholders prioritise quality and sustainability, serving as custodians of heritage [9]. Meanwhile, hobbyists contribute to local food systems and strengthen community bonds through activities like beekeeping [10]. Moreover, entrepreneurial smallholders seize opportunities in niche markets, offering artisanal products and using social media as a marketing tool [11]. On the other hand, the urban group strives to transform city areas into hubs of food security and environmental sustainability [12]. Nature-connected lifestyles are integrated with farming by homesteaders, while Scottish crofters in remote areas proudly uphold communal grazing and their unique cultural heritage [13].

The need for efficient and thorough surveillance of these farmers in the UK has grown more urgent in recent years as the agricultural industry deals with mounting pressures from climate change, shifting consumer demands, and regulatory requirements [14]. However, because of the particular features of these farming systems, implementing such surveillance mechanisms has proven to be a complex task. Establishing a unified and methodical approach to surveillance is significantly hampered by the heterogeneous nature of these farms in terms of their dimensions, areas of production concentration, and management techniques [15]. Notably, the monitoring environment is further complicated because small-scale poultry keepers with fewer than fifty birds are not required to register their flock [6].

With the rise of social media platforms like Twitter, it is now feasible to investigate niche networks, identify prominent members, and find communities in real time [16]. Twitter, in particular, has emerged as a vital source of information, offering insights into numerous elements of agricultural and animal health [17]. In this context, the current study aims to examine smallholder farming networks, identify significant individuals, and find communities inside the networks using Twitter data.

The community structure of smallholder livestock farmers, veterinarians, and other stakeholders involved in animal health plays a crucial role in the surveillance and control of animal diseases [18]. Key aspects of community structure and their implications for animal disease surveillance and control include information sharing and communication, adoption of best practices, collective action, disease monitoring and reporting, tailoring interventions, and reducing barriers to collaboration [19].

Communities with strong social networks and open communication channels are better equipped to share information on disease outbreaks, best practices, and control measures, enabling faster responses to emerging threats and a more effective dissemination of crucial knowledge and resources [20]. Those networks with a strong sense of cohesion and trust are more likely to engage in collective action to address animal health issues. Monitoring and reporting diseases can enable the tracking and reporting of outbreaks, tailoring interventions to the specific needs, resources, and dynamics of communities, and reducing obstacles to collaboration.

Scale-free networks are commonly found in various real-life networks and are characterised by a small number of nodes that have a large number of connections, acting as hubs [21].

The hub nodes differ significantly from most nodes, which have a relatively low level of connectivity. The term "scale-free" is used because this property leads to a self-similar node degree heterogeneity across scales [22]. This phenomenon is present in different types of real-world networks, such as social systems, biological networks, and the Internet, emphasising its importance in comprehending networked systems [23].

### 1.1 Study aims

The study's aims are threefold. First, we will use Twitter data to learn more about smallholder farming networks and how they interact. Second, we want to find prominent users inside these networks who can help spread information and shape animal health practices. Finally, we will use community identification methods to investigate the underlying structure of smallholder networks and how these communities may influence animal health management.

By fulfilling these goals, our research will considerably contribute to our understanding of smallholder farming networks, their connections, and concerns. Furthermore, our findings will be useful to policymakers, industry stakeholders, and researchers in developing more effective techniques for disease detection, control, and overall animal health improvement in smallholder farming communities.

### 1.2 Related work

Numerous studies have focused on smallholder farming, emphasising its importance in global food security and rural livelihoods [1]. The link between smallholder livestock farmers and animal health is well-established, with several studies highlighting the necessity of effective animal health management methods for long-term production and food safety [24,25]. Disease prevention, control, and surveillance are critical for smallholder livestock farmers to preserve productivity and animal welfare [26].

Network analysis within animal health has developed as an effective method for investigating the structure and dynamics of agricultural communities [5,16]. Several studies have used network analysis to better analyse farmer relationships, interactions, and information flow. This research has revealed that network properties such as centrality and clustering play an important influence in knowledge transmission and the adoption of effective practices [16]. In the area of animal health, network analysis has been used to explore disease spread and evaluate the efficacy of control strategies [27].

The advent of social media platforms has opened a new window into human behavior and interactions. Due to their ability to change ideas and drive trends, influential users in social media networks have been a topic of interest [28]. Several approaches for identifying prominent users have been developed, including the use of centrality measurements, user interaction, and content analysis [29]. Simultaneously, community detection algorithms have been used to discover the underlying structure of social media networks and identify groups of people with shared interests or communication habits [30].

Our paper discusses the intersection of smallholder farming, network analysis, and social media, but we must consider related research in other fields. Network analysis and social media have been widely researched in the realm of human health, offering valuable perspectives on disease transmission, health communication, and behavioural changes [18]. Nevertheless, the literature on livestock and animal health is considerably less abundant. Although there are stand-alone studies on small-scale farming and animal health, and separately on network analysis and social media, there are few that examine the overlap of these fields. The growing significance of social media as a communication tool and the critical role of smallholder livestock farmers in food security and disease outbreak containment highlight the need for further research in this domain. We aim to bridge this gap by exploring the potential of social media mining and network analysis to better understand the interactions between this demographic.

## 2 Methodology

### 2.1 Data privacy, informed consent and ethics

In compliance with Twitter's Data Use Policy and local data protection laws, all data were collected and used. Although individual user consent was not obtained, the collected data is public and permitted by Twitter's terms of service. Our research involved the extraction of user IDs, locations, and profile descriptions, which were later anonymised to create the final dataset. We did not access or use any private user data in our study. Our data collection and analysis processes prioritise user privacy and confidentiality.

Developer level access was permitted by Twitter and obtained in October 2019, granting administrative permission to access the raw twitter data. All data was anonymised prior to analysis. No user identifiable data was scraped, and all text was aggregated and analysed together, hence no individual can be identified from the results.

Ethical approval was granted by the University of Stirling's General University Ethics Panel (GUEP) and conformed to the research integrity policies. All methods were carried out in accordance with relevant guidelines and regulations surrounding social media scraping and analysis provided by UK research and Innovation (UKRI).

### 2.2 Anonymisation of users

To protect user privacy, we anonymised the user IDs in the network by creating unique numeric identifiers for each user. We then mapped this anonymised data to the original IDs, ensuring that it protected the privacy of the users within the network throughout the analysis, in addition to having obtained prior ethical approval for the use of this data and subsequent analysis.

### 2.3 Data collection

To collect data from Twitter, we used the Tweepy library in Python, which provides a convenient interface for accessing the Twitter API [31]. Developer-level access was obtained prior to analysis, allowing for a greater degree of data extraction through the Twitter API. Followers were scraped from a large smallholding twitter account (@SmallholdersUK) generating a database of over 20,000 users from within the UK. User IDs (which were later anonymised), locations and profile descriptions were extracted to build the final dataset.

### 2.4 Sampling approach

Due to our emphasis on smallholder livestock farmers in the UK and Twitter being our primary data source, we adopted purposive sampling, a non-probability approach, for our sampling strategy [32].

The justification for choosing to extract data from the @SmallholdersUK Twitter account was based on its representation as the largest online community dedicated to smallholder farming in the UK. Its substantial follower base provides a rich and extensive data set, which increases the likelihood of capturing a representative and comprehensive picture of the community and allows for sufficient network analysis to be conducted.

### 2.5 Contextualising the study population

In order to provide context for our findings, it's essential to comprehend the smallholder landscape in the UK before continuing with the analysis.

In the UK, small scale farms are known for their sustainable and diversified operations. Typically, these enterprises are run by families and oversee a relatively small plot of land. These farmers can raise different livestock species, ranging from pigs, cattle, poultry and even bees. The varied farming practices can highlight the diversity within this demographic, as some individuals may adopt organic or free-range farming methods, whilst others remain with more traditional methods. The farmers usually sell their produce directly to customers at local farmers' markets or community-supported agriculture schemes.

Our study population comprises the followers of the @SmallholdersUK Twitter account, but these traits may not completely define them. This online community might show distinctive characteristics and practices that set them apart from the typical smallholder farmers, however a greater level of demographic data is required to make such inferences. Twitter alone may not capture all the relevant demographic information of this cohort, and additional secondary sources such as smallholding forums may provide a more holistic picture. Niche forums can capture a wider array of information pertaining to numbers and types of livestock, which can be used as a supplement to this Twitter analysis.

### 2.6 Network analysis

We began by retrieving the target user's list of followers and subsequently forming a network of links between the smallholder and their followers.

A dataset was constructed with two columns, ‘source' and 'target'. The ‘source' column represents the user being followed, while the 'target' column represents the source users’ followers. In the context of our study, the source user is the smallholder farmer whose network we are interested in analysing.

The network data collected was then used to create a graph using the NetworkX library [33]. We created a directed graph to represent the Twitter follower network, with nodes representing users and edges representing follower relationships.

Due to the computational and temporal costs of huge networks, and in order to produce a more focused representation, we limited the number of nodes presented in the network to the top 50 nodes based on their degree (i.e., the number of connections each node has).

### 2.7 Network metrics

Several metrics were applied to the network graph, including degree centrality, betweenness centrality, closeness centrality, and modularity [34]. These metrics give information on the smallholder network's structure and features.

### 2.8 Influential user identification

We computed four alternative centrality metrics for each node in the graph to identify significant people inside the smallholder livestock farmers' Twitter network: degree centrality, betweenness centrality, eigenvector centrality, and PageRank [34]. The nodes were sorted based on their centrality values, and the top 10 nodes for each centrality metric were obtained.

Finally, for each centrality metric, we generated a dataset that held the user IDs of the top ten important users, allowing us to compare the findings and identify prominent players in the network based on different criteria.

### 2.9 Text pre-processing

The study used natural language processing techniques to clean and pre-process the text data, for the purposes of user classification. Furthermore, custom stop words were integrated to eliminate data noise.

The text cleaning function cleaned and standardised the text prior to performing the analysis. URLs, mentions, and hashtags were removed, text was converted to lowercase, punctuation marks were removed, and the English translate functions were deployed to clean up the text (user profile descriptions and timelines).

User profile descriptions and timelines were combined into a singular column prior to performing text categorisation, and any rows which observed an empty string were subsequently removed. The frequency of the raw data was 20658 and after processing, this was reduced to 17725.

### 2.10 User categorisation

We adopted a keyword-based categorisation approach to categorise users based on their profile description and user timelines. The methodology included the following steps:

A dictionary of categories was created as displayed in Table 1, containing several user categories as keys (e.g., "smallholder", "academic", "veterinary", "farmer", "individuals", "crofter", "other"), and their corresponding lists of keywords as values.

**Table 1:** Manually coded user categories with corresponding keyword matching terms.

Categories	Matching Terms
Smallholder	["smallholder", "small farm", "small-scale farmer", "family farm"]
Academic	["professor", "researcher", "lecturer", "academic", "scientist", "scholar"]
Veterinary	["veterinarian", "vet", "veterinary", "animal health"]
Farmer	["farmer", "agriculture", "farming", "agronomist", "rancher"]
Individuals	["enthusiast", "hobbyist", "freelancer", "bird lover", "poultry keeper"]
Crofter	["crofter", "crofting", "croft farm", "croft farmer"]
Other	[]

This methodology enabled the classification of users in the network according to their descriptions, facilitating further analysis of the network structure and user interactions based on these categories. This approach can be adapted to other keyword-based categorisation tasks and provides a straightforward method for classifying textual data based on predefined criteria.

### 2.11 Community detection

By visualising the follower network and detecting communities, we were able to gain insights into the structure of the network and identify distinct groups within the community, as similar studies have also performed [30]. This consisted of both modularity-based algorithms and hierarchical clustering methods. The main justification for using both approaches is because they provide different viewpoints on the network's basic structure. Hierarchical clustering techniques construct a dendrogram to describe the nested relationships between clusters, whereas modularity-based algorithms maximise a modularity score to discover well-separated communities. By combining the two methods, researchers may assess the resilience of the detected communities and get a more thorough knowledge of the network's community structure. Also, comparing the outcomes of these two approaches may aid in validating the conclusions and guarantee a more trustworthy and correct interpretation of the data.

#### 2.11.1 Modularity-based algorithms

We employed the Louvain method for community detection, which is known for its efficiency and accuracy in uncovering communities in large networks [35]. After computing the best partition, we visualised the network with nodes colored by community.

We sought to study the distribution of communities and their frequency within the network after discovering them using the Louvain approach. To do this, we developed a dataset to contain the partition information, with each row representing a user and the community to which they belong.

#### 2.11.2 Hierarchical clustering methods

We used text vectorization, dimensionality reduction, and hierarchical clustering techniques to analyse and group users based on their textual descriptions.

Text Vectorization was achieved through converting the text data into numerical features. This was done using the Term Frequency-Inverse Document Frequency (TF-IDF) method [36], effectively capturing the importance of words within the user descriptions while accounting for their frequency in the entire dataset. Given the large network of followers, we employed dimensionality reduction using the singular value decomposition (SVD) technique, which aids in facilitating a more efficient clustering analysis in subsequent steps [37].

The next step involved calculating a distance matrix based on the cosine similarity of the reduced-dimension data. This matrix represented the pair-wise distances between data points, capturing the dissimilarity between user descriptions. This matrix was used as the input for the clustering algorithm, Agglomerative Hierarchical Clustering using the Ward Linkage. This allowed the identification of patterns and the ability to group users accordingly.

A scatter plot of the t-distributed Stochastic Neighbor Embedding (t-SNE) was created to visualise the high-dimensional data to show the results of the clustering analysis on the textual dataset. The feature space was dimensionally reduced to 100 components using Truncated Singular Value Decomposition (Truncated SVD) before using t-SNE. The dimensions were then further reduced to 2 using t-SNE, allowing for the display of the data points in a two-dimensional environment.

Hierarchical clustering was applied to identify groups of users with similar descriptions, followed by the ward method to minimise the sum of squared differences within clusters, and this generated a hierarchical representation of the relationships between users.

### 2.12 Geolocation mapping

To aid the spatial visualisations, we plotted the locations of the Twitter profiles in the network to analyse the spatial distributions. As the extracted profiles contained a free-text location column, the “Geopy” package was utilised to convert the locations to numerical co-ordinates, which were subsequently plotted on a heat map, as seen in Fig. 6.

## 3 3/. Results

**Fig. 1:**
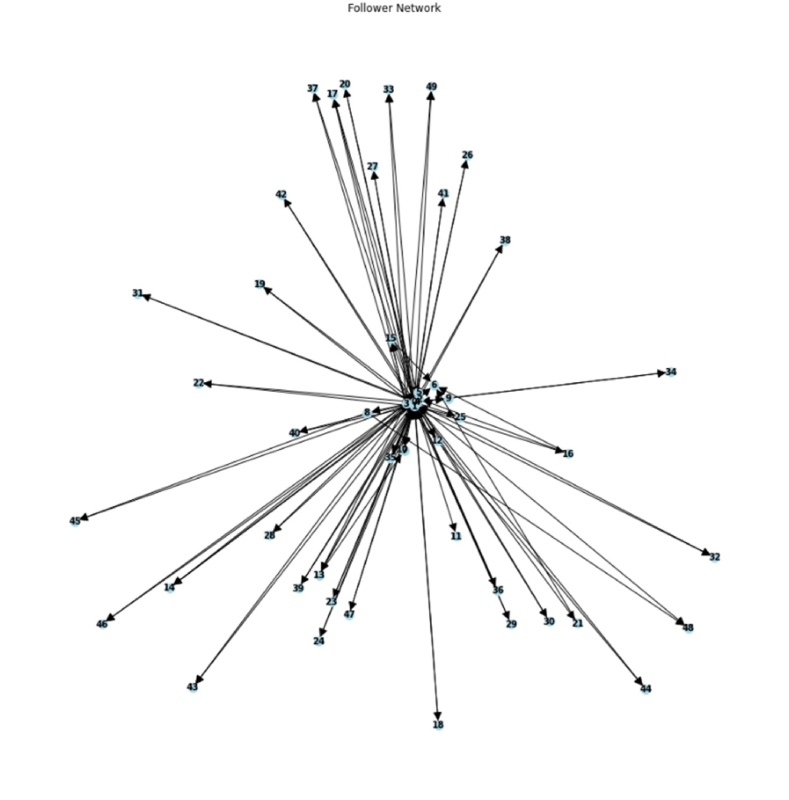
Network visualisation of user interactions highlighting key nodes and connections.

The examination of the follower network in Fig. 1 yielded a number of intriguing patterns and insights into the dynamics and structure of the network.

### 3.1 Overview of the smallholder network

As the follower network was visualised, it was evident that it had a scale-free topology, with a small number of hubs that had many connections and a huge number of nodes with far fewer connections. Many users were linked to these hubs directly or via intermediary nodes, serving as the network's hubs. This discovery was in line with the features of social networks, where a select group of powerful users frequently has a big influence on the general network structure.

### 3.2 Influential users and their roles in the network

The top three phrases connected to distinct anonymised IDs were examined using four different centrality methods, including degree, between, and eigenvector centrality.

The most frequent category in the degree centrality analysis (Table 2) was "other," which included a wide range of subjects including employment, nature, and personal interests. A smallholder was the most influential user according to this measure. There were a few academic IDs that concentrated on health and newsletters as well. In contrast, Table 3's between centrality analysis revealed a more varied collection of categories with a stronger prevalence of academic and veterinary topics. Leading keywords in this table included words related to newsletters, energy, and animals.

**Table 2:** Degree centrality measure results displaying the ten highest ranked users, along with the top 3 terms found in their user timeline, and corresponding classification category.

Anonymised ID	Top 3 Terms	Category
0	['grass', 'video', 'haylage’]	smallholder
3	['day', 'read', 'medium’]	other
8	['please', 'see', 'health’]	academic
9	['love', 'would', 'like’]	other
10	['know', 'sperm', 'whale']	other
11	['thing', 'health', 'day’]	academic
12	['work', 'consultant', 'junior’]	other
13	['plant', 'wildflowerhour', 'spring']	other
14	['green', 'need', 'party’]	other
15	['nature', 'dawn', 'chorus']	other

**Table 3:** Between centrality measure results displaying the ten highest ranked users, along with the top 3 terms found in their user timeline, and corresponding classification category.

Anonymised ID	Top 3 Terms	Category
103	['elephant', 'asian', 'stae']	veterinary
57	['energy', 'year', 'new']	academic
92	['time', 'god', 'tide']	other
59	['folk', 'horror', 'bird']	other
84	['thanks', 'thank', 'ardnamurchan']	other
17	['newsletter', 'substack', 'note']	academic
100	['yes', 'haha', 'yorkshire']	other
16	['blossom', 'tulip', 'cherry']	other
3	['day', 'read', 'medium']	other
58	['puppy', 'pet', 'great']	other

The bulk of the IDs in the eigenvector centrality study (Table 4) were labelled as "other," however several IDs were classed as "veterinary.", the most out of any of the other measures. Findings from the NLP analysis included phrases for personal hobbies, patriotism, and social media.

**Table 4:** Eigenvector centrality measure results displaying the ten highest ranked users, along with the top 3 terms found in their user timeline, and corresponding classification category.

Anonymised ID	Top 3 Terms	Category
24487	['amp', 'kennedy', 'want']	other
24488	['follow', 'account', 'drop']	other
24491	['usmc', 'veteran', 'patriot']	veterinary
24492	['amp', 'bill', 'three']	other
24493	['video', 'tiktok', 'check']	veterinary
24494	['happy', 'love', 'friday']	other
24495	['order', 'link', 'good']	other
24496	['marxist', 'care', 'dear']	other
24497	['let', 'get', 'disloyal']	veterinary
24498	['god', 'ceo', 'buying']	other

Just one veterinarian ID was found in the PageRank analysis (Table 5), where the majority of IDs were once again labelled as "other." The topics in this table varied from politics and conflict to hobbies and health.

**Table 5:** Page rank measure results displaying the ten highest ranked users, along with the top 3 terms found in their user timeline, and corresponding classification category.

Anonymised ID	Top 3 Terms	Category
21845	['government', 'lying', 'number']	other
21846	['borrowdale', 'stay', 'award']	other
21847	['war', 'amp', 'asking']	other
21848	['rescue', 'mountain', 'men']	other
21850	['great', 'lakedistrict', 'easter']	other
21715	['youtube', 'full', 'amp']	other
21716	['pay', 'people', 'rise']	other
21720	['metabolic', 'health', 'symposium']	veterinary

A wide range of themes were disclosed by these multiple centrality metrics, with the majority of anonymised IDs falling into the "other" group. Although while the veterinary and academic categories were less prevalent, they nonetheless offered insightful information about certain interests and themes in the dataset. Of the several influential metrics, Anonymised ID 3 appeared the most frequently, twice in Table 2, Table 3

Unconventional observations were discovered among the most influential users in the between centrality rank of Table 3, including terms like "elephant," "Whale," and "Ardamurchan." In the context of smallholder farming, the inclusion of terms such as "elephant", "whale", and "Ardnamurchan" among the most influential users may indicate the importance of these topics within the farming community discussions. Although not typically associated with smallholder livestock farming, "elephant" and "whale" may represent a specific set of health issues related to larger animals that could be significant in specific contexts or regions. The allusion to these animals could also represent wider ecological or environmental problems that affect small-scale agriculture. It's possible that the usage of "Ardnamurchan" implies the importance of issues affecting the local smallholder livestock farmers in that region of Scotland. The reason for this could be distinctive farming methods, regional animal health problems, or governmental regulations implemented in this location.

### 3.3 Community detection

**Fig. 2:**
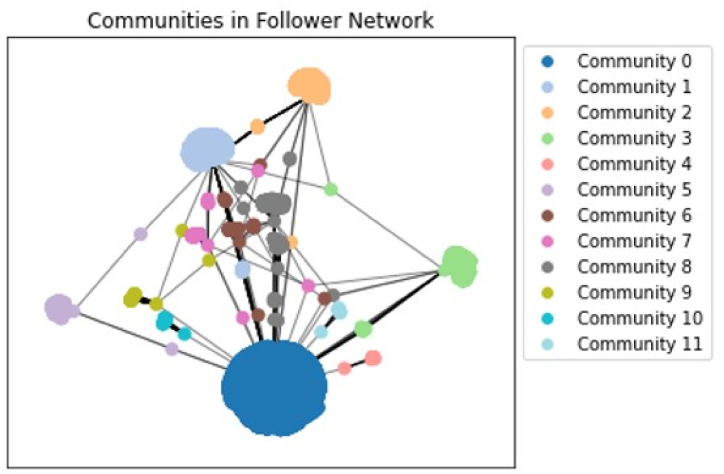
Community detection network visualisation depicting the relative size and connections between communities.

The results from the community detection is depicted in Fig. 2 offer important new information about the organisation and connections between network members.

**Fig. 3:**
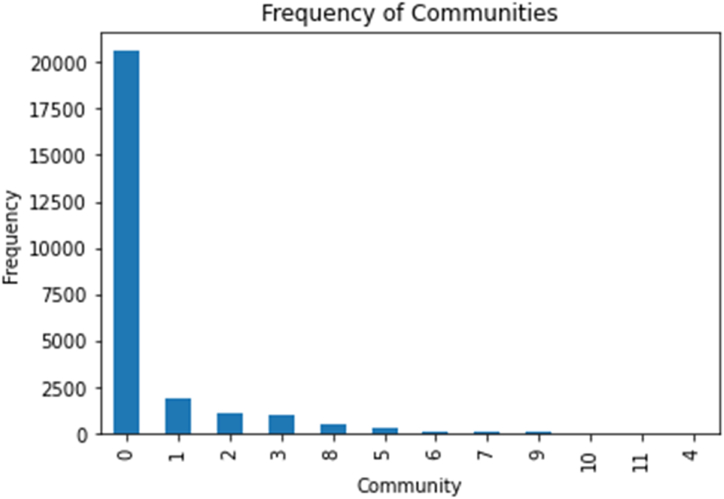
Bar graph showing the frequency distribution of the communities.

Based on the connections between individuals, the community detection algorithm discovered 11 communities, with a strong right skew visualised in Fig. 3. Community 0 represents the largest network of followers as it is the largest smallholding Twitter account. The rest of the communities all yield a frequency fewer than 2500, with communities 1,2 and 3 being the largest amongst them. These communities reflect groups of individuals who have common links or interests, which can be useful for comprehending the network's underlying dynamics.

**Fig. 4:**
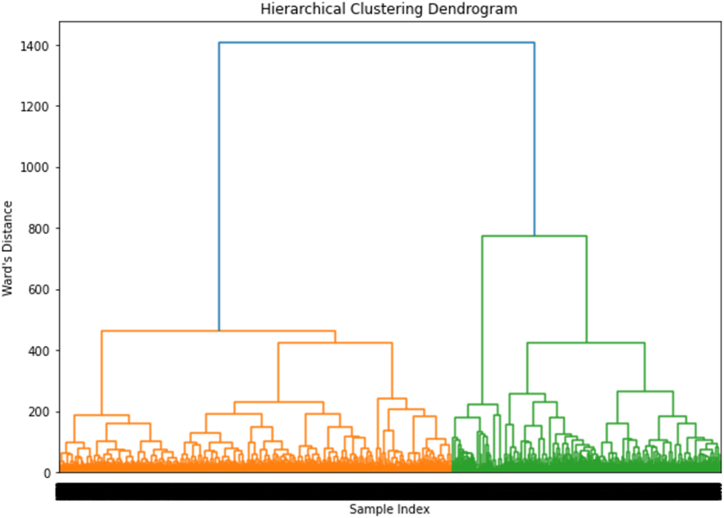
Hierarchical clustering dendrogram: The dendrogram displays data points linked into clusters based on similarity (Ward's distance). The x-axis shows the sample index; the y-axis indicates the merge distance. Larger lines suggest greater dissimilarity between merged clusters. This helps identify the optimal cluster count for the data.

Fig. 4 displays the dendrogram of the clustering hierarchy and the outcomes of the hierarchical clustering analysis performed on the textual dataset. The feature space was reduced to 100 components using dimensionality reduction using the SVD method.

Furthermore, it illustrates the data's hierarchical structure, with each branch signifying the union of two clusters depending on their degree of similarity. Ward's distance is shown on the dendrogram's y-axis. Clusters are combined based on a greater degree of resemblance as we advance up the y-axis. We chose an acceptable cut-off point for grouping based on the dendrogram's visual examination, resulting in three different clusters (n = 3). The cut-off point was used to increase cluster distance while decreasing intra-cluster variation. The longest vertical line in the dendrogram that was not crossed by any lengthy horizontal lines served as the basis for the choice.

In addition, further examination was undertaken relating to the distribution of the data points among the three clusters after applying cluster labels to the dataset. As seen in Table 6, 10544 data points make up Cluster 1, 1376 make up Cluster 2 and 5805 data points make up Cluster 3. According to these outcomes, the clustering algorithm effectively divided the textual data into three different groups, producing a useful dataset partition based on the similarity of the feature vectors in the condensed space. To understand the underlying themes or issues that define each cluster, further analysis of the material inside each cluster may be done.

**Table 6:** Distribution of frequencies across clusters from the hierarchical clustering analysis.

Cluster	Frequency
1	10544
2	1376
3	5805

**Fig. 5:**
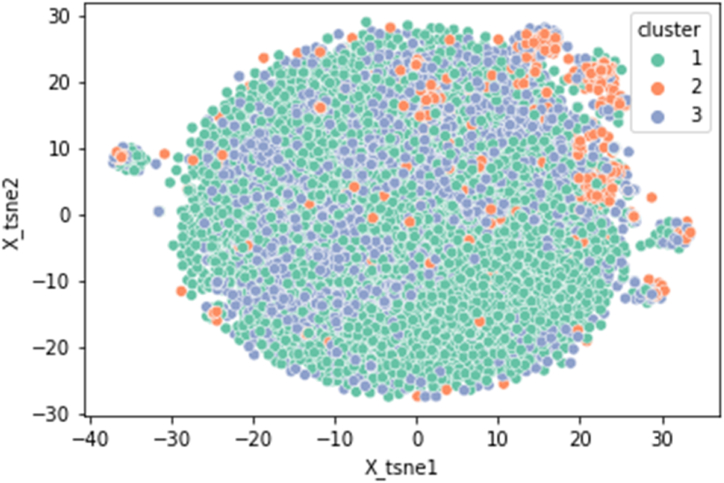
T-distributed Stochastic Neighbor Embedding (t-SNE) visualisation: Data points are segmented into three distinct clusters. The axes, labelled 'x_tsne1' and 'x_tsne2,' represent the two-dimensional space resulting from the t-SNE reduction.

Fig. 5 presents a scatter plot of the t-distributed Stochastic Neighbor Embedding (t-SNE) visualisation of the high-dimensional data to show the results of the clustering analysis on the textual dataset.

**Fig. 6:**
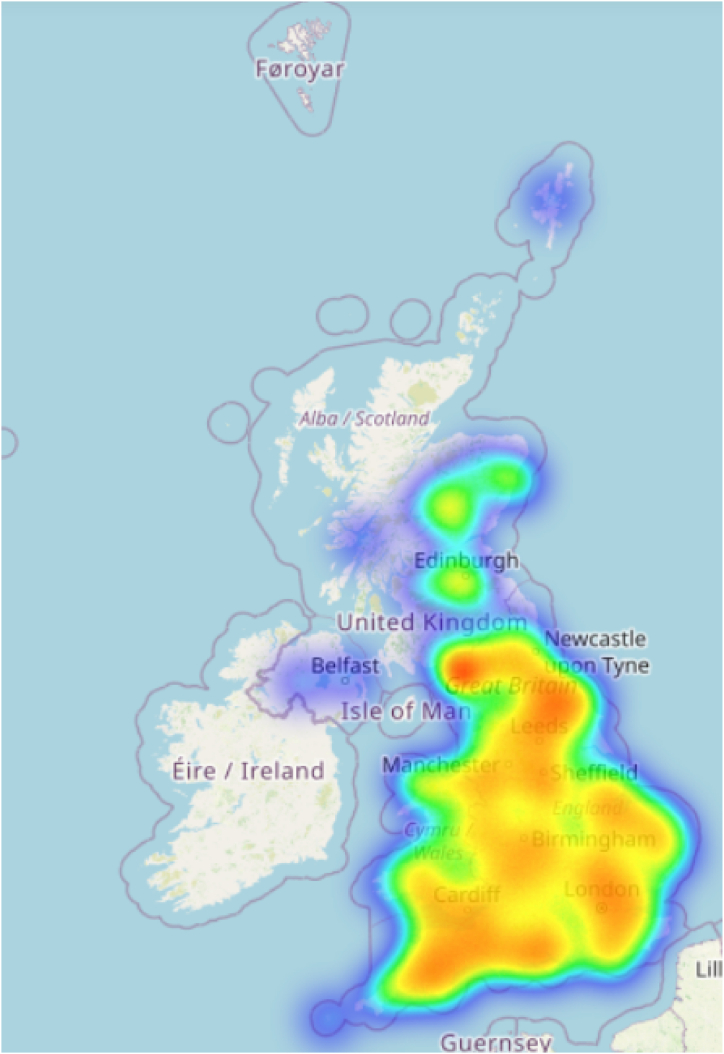
Heatmap of UK locations: The intensity of colour denotes the concentration of user locations, with warmer colours indicating greater levels of population prevalence.

Each point is a data sample, and its colour reflects the cluster to which it belongs. The scatter plot demonstrates how the clusters are divided in the two-dimensional t-SNE space and provides information on how the data points are distributed throughout the clusters. Due to the closeness of the feature vectors in the t-SNE space, the clustering algorithm was able to successfully divide the textual data into three separate groups, producing a useful dataset division. Cluster 1 was the dominant group, covering the majority of the data points in a uniform manner, whereas cluster 2 was sparser and more concentrated in the top right-hand side of the plot.

The scatter plot's ability to separate the clusters indicates how well the dimensionality reduction methods worked.

### 3.4 Geolocation mapping

The spatial distribution of the studied locations across the United Kingdom were visualised using a heatmap generated with the folium library in Python, as depicted in Fig. 6. The heatmap was based on the latitude, longitude, and count (if available) of each location within the UK's geographical boundaries, which were defined by the following latitude and longitude bounds: minimum latitude of 49.8, maximum latitude of 60.9, minimum longitude of −10.9, and maximum longitude of 2.0.

The heatmap displayed a colour gradient representing the density of the locations, with darker shades indicating higher concentrations and lighter shades representing lower concentrations. The radius parameter was set to 15, which determined the size of the circular areas around each data point, with larger values corresponding to wider areas of influence.

Higher concentrations of locations were predominantly found in urban areas such as London, Manchester, Birmingham, Glasgow, and Edinburgh. These urban centres displayed the darkest shades on the heatmap, indicating a significantly higher density of locations compared to other regions.

In contrast, rural and remote areas, particularly in the Scottish Highlands, western Wales, and parts of southwest England, exhibited lighter shades on the heatmap, suggesting a lower density of locations. The general trend observed was a decrease in location density as the distance from urban centres increased, with the exception of a few notable clusters of locations in smaller towns and along major transportation routes.

The results of this heatmap analysis provide valuable insights into the spatial distributions of smallholder livestock farmers and their followers.

## 4 Discussion

### 4.1 Interpretation of the results in the context of smallholder livestock farming in the UK

The analysis showed that a vast majority of the anonymous IDs were assigned to the 'other' classification. This observation indicates that the sample employed in the study may not represent the diverse segments in the agricultural community, as it appears to be skewed towards non-farming aspects. A lack of sufficient focus or consideration could be impacting smallholder farming. Ensuring adequate representation of smallholder farming is crucial in future studies since they are a critical component of agriculture. The discovery of diverse communities inside the network shows the existence of multiple user groups with related links or interests, which may be a sign of specialised farming methods, local agricultural trends, or common experiences among farmers. It is possible to better meet the unique needs and concerns of these groups via modifying communication techniques and actions with the aid of an understanding of these groups.

Fronczak (2018) discovered that the scale-free topology is still prominent in modern online social networks such as Facebook and Instagram [21], which is line with the structure of the follower network in Fig. 1.

Furthermore, the role of influential users in affecting public opinion and sharing information on social media platforms such as Twitter has been extensively researched [38]. Cha et al. (2010), for example, evaluated the influence of Twitter users by assessing several centrality measures, which is comparable to the methodologies utilised in the current stud, and emphasized the role of a few key players in information dispersal amongst the communities.

Fortunato and Hric (2016) underlined the importance of community detection in complex networks, particularly for understanding the structure and function of social networks [39]. Their work reveals how community detection may be used on a variety of modern network datasets, including cooperation networks, citation networks, and online social networks. In the context of this analysis, eleven communities were derived from the results, and further analysis into the demographics into each communities would provide an in-depth examination of the unique attributes and similarities between each community.

### 4.2 The potential impact of influential users on information dissemination and animal health practices

Throughout the analysis, we identified influential users, particularly from academic and veterinary backgrounds, as crucial drivers in disseminating accurate and reliable information on animal health procedures among smallholder farming groups. Although fewer in number, these important individuals can motivate the implementation of proper hygiene practices, resulting in better animal health outcomes.

. By analysing centrality measures, we were able to pinpoint influential users who can significantly impact information dissemination and the uptake of best practices in animal health management. Engaging with these users and leveraging their networks can amplify the effectiveness of animal health campaigns and promote better farming practices among smallholder livestock farmers. However, due to the limited size of these identified cohorts, it's important to note that our conclusions about the overall impact may be constrained.

### 4.3 Impact on society and the environment

Identifying clusters of smallholder farmers in urban planning can assist in infrastructure development. For instance, it can help in planning transportation routes, optimising supply chain, and enhancing market access. Additionally, this information can help public health officials pinpoint areas with a high concentration of livestock for disease surveillance and prevention. Effective health risk management and targeted disease control strategies can be implemented by understanding the locations of large networks, and thus expediting the process of disease containment.

The location of livestock can impact the environment both positively and negatively for environmental management. One instance is the contribution of livestock to biodiversity in some regions, yet in other regions, it may cause overgrazing and degradation of habitats.

These findings can also be instrumental in supporting initiatives to decrease carbon emissions in agriculture, which is a fundamental aspect of the UK's Net Zero Growth Plan [40]. Methane, which is a potent greenhouse gas, is significantly released by livestock farming, particularly intensive methods. Better understanding of the distribution and scale of smallholder farming can enhance our ability to estimate agricultural emissions and pinpoint potential intervention areas.

### 4.4 Limitations of the study and future research directions

The research examined the dynamics of smallholder livestock farmers in the context of online social networks. It was based on extracting the followers of one particular account, which might not fully reflect the complete population of smallholders, or the full spectrum of themes covered. Moreover, the analysis focused on centrality metrics that could not accurately reflect all facets of influence and information flow in these groups.

Future research should consider more substantial and representative sample sizes, as well as a wider variety of social network analytic approaches. The community detection analysis conducted on the given network provides valuable insights into the structure and relationships among users in the context of smallholder farming, however it is not without its limitations. A more thorough implementation of hyper tuning the parameters with the two community detection models needs to be applied, backed by a robust custom stop word dictionary which can alleviate the majority of those noise found with the textual input.

The insights gained through the analysis provided several key revelations, which can inform targeted interventions and communication strategies to improve animal health practices and control the spread of diseases. However, the data used in the study is based on social media interactions, which may not fully represent the broader population of smallholder livestock farmers.

Although our research has focused mainly on smallholder farming in the UK, it's crucial to consider the wider global context of smallholder farming. Developing regions like West Africa may have different smallholder farming systems due to factors such as internet access, social media platforms, and cultural practices [4]. Nevertheless, the pivotal role of influential individuals in disseminating precise information may be a universal factor across diverse regions.

The structure and relationships observed among smallholder farming communities online could be similar in these areas. This reinforces the requirement of parallel studies which compare the communities throughout various regional contexts, including both developing and developed nations.

Finally, additional research could expand the analysis to include other data sources, such as interviews or surveys, and incorporate additional context to better understand the relationships and dynamics within and between communities. Finally, further research is needed to refine the understanding of these communities and their unique characteristics, and to evaluate the effectiveness of interventions leveraging the identified community structures and influential users. One possible way this could be achieved may arise through incorporating the sentiments and opinions of these communities based on tweets by livestock authorities, namely The Animal and Plant Health Agency (APHA), in addition to messages disseminated by veterinarians.

## 5 Conclusion

We conducted a comprehensive network analysis of smallholder livestock farmers using social media data, particularly from Twitter, in this study. We were able to deeply explore the interactions within this group using this approach. Through our analysis, we discovered significant insights, such as identifying influential users who have the potential to shape conversations and practices within the smallholder farming community due to their reach and engagement levels. We identified separate community structures in the network, revealing how small-scale farmers are linked, interact, and exchange information.

More significantly, these insights come from more than just smallholders. This has the potential to improve disease outbreak management and speed up the spread of crucial information among smallholder livestock farmers. Our usage of social media data in network analysis improves our comprehension of this community and uncovers feasible ways to enhance their livestock health practices and interactions with stakeholders.

The gathered results of this paper have various policy, industry, and research ramifications. Knowing the network structure and key users can assist policymakers and organisations such as APHA in tailoring their communication methods to guarantee successful transmission of animal health information. It also enables for more precise targeting of surveillance and control operations, with an emphasis on high-risk areas or people (which can be visualised through geolocation mapping as seen in Fig. 6), potentially improving overall health outcomes for smallholder farming communities.

This knowledge can be used to benefit industry stakeholders (i.e. research institutions, agricultural supply companies, farmer's cooperatives etc) by interacting with prominent users and communities to promote best practises, goods, and services suited to the specific needs of smallholder livestock farmers. Furthermore, the observed conclusions can be used to better investigate the dynamics of smallholder networks and their implications for animal health and disease management.

In the context of social media as a research tool, his paper has outlined encouraging findings on the use of social media data in smallholder agricultural research. Social media platforms are an important source of information for comprehending the dynamics of smallholder livestock farming communities as they continue to gain popularity and become more integrated into daily life. Future studies can make use of social media data to investigate several other facets, including information exchange, farmer behaviour, and the uptake of new technologies.

However, it also necessitates careful attention to ethical considerations. Among the most important considerations for data privacy are adhering to platform-specific policies, obtaining informed consent, and anonymisation. Self-reported data may contain inaccuracies, and social media users may not be representative of this farming population in general. Additional factors have to be considered, such as individuals refraining from adopting social media in favour of more traditional networking avenues, namely smallholding events.

In conclusion, a valuable understanding of the structure, important users, and community dynamics within this population has been gained through this research. These findings show the possibility of using social media data to better understand these farming systems and address the issues encountered by these communities, and they have significant ramifications for policy, the environment, business, and research.

## Ethics statement

Developer level access was permitted by Twitter and obtained in October 2019, granting administrative permission to access the raw twitter data. All data was anonymised prior to analysis. No user identifiable data was scraped, and all text was aggregated and analysed together, hence no individual can be identified from the results. Ethical approval was granted by the University of Stirling's General University Ethics Panel (GUEP) and conformed to the research integrity policies.

All methods were carried out in accordance with relevant guidelines and regulations surrounding social media scraping and analysis provided by UK research and Innovation (UKRI). Further information can be found here: https://www.ukri.org/councils/esrc/guidance-for-applicants/research-ethics-guidance/internet-mediated-research/

## Informed consent

The need for informed consent was waived by the University of Stirling's General University Ethics Panel (GUEP).

## Consent for publication

Not applicable.

## Funding

This research did not receive any specific grant from funding agencies in the public, commercial, or not-for-profit sectors.

## Data availability

Data will be made available on request. The data has not yet been submitted into a publicly available repository however the data will be made publicly available upon the completion of the PhD examination at the following GitHub repository: https://github.com/a-s-munaf. All data can be reproduced via the Twitter API application, following the methodology purported in this paper.

## CRediT authorship contribution statement

**Samuel Munaf:** Writing – review & editing, Writing – original draft, Visualization, Software, Resources, Methodology, Investigation, Formal analysis, Data curation, Conceptualization. **Kevin Swingler:** Writing – review & editing, Validation, Supervision, Project administration, Methodology, Investigation, Conceptualization. **Franz Brulisauer:** Conceptualization, Project administration, Supervision, Writing – original draft. **Anthony O'Hare:** Conceptualization, Project administration, Supervision, Validation, Writing – original draft. **George Gunn:** Conceptualization, Supervision, Writing – original draft. **Aaron Reeves:** Conceptualization, Investigation, Methodology, Project administration, Resources, Supervision, Writing – original draft, Writing – review & editing.

## Declaration of competing interest

The authors declare that they have no known competing financial interests or personal relationships that could have appeared to influence the work reported in this paper.
